# Disodium calcium dinickel(II) bis­[diphosphate(V)] deca­hydrate

**DOI:** 10.1107/S160053681100016X

**Published:** 2011-01-15

**Authors:** Yun-Cheng Cui, Yan-Hui Zhao

**Affiliations:** a Department of Chemistry, Jilin Normal University, Siping, Jilin 136000, People’s Republic of China

## Abstract

In the title compound, Na_2_CaNi_2_(P_2_O_7_)_2_(H_2_O)_10_, there are two distinct P-atom sites, each tetra­hedrally coordinated by four O atoms. The resulting phosphate tetra­hedra link through a common O atom, forming a [P_2_O_7_]^4−^ diphosphate unit. The Ni—O coordination is square pyramidal with four O atoms from two diphosphate groups in equatorial positions and the vertex occupied by a water O atom. The (P_2_O_7_)(H_2_O) units link the Ni atoms, forming a chain of pyramids and tetra­hedra. As a result of the *d*-glide and twofold-axis symmetry of space group *Fdd*2, the chains propagate along [101] and [10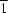], and chains in adjacent layers are mutually orthogonal. The Ca cation, located on a rotation axis, and the Na cation are each octa­hedrally coordinated by four O atoms and two waters. The Ni-chain arrangement is stabilized by Ca and Na coordination and a network of O—H⋯O hydrogen bonds.

## Related literature

For the isotypic copper(II)–diphosphate mineral wooldridge­ite, see: Cooper & Hawthorne (1999 ▶). For the structure and magnetic properties of a diphosphate-bridged Cu^II^ complex, see: Kruger *et al.* (2001 ▶). For two other rare examples of Ni^II^ and Co^II^ coordination complexes with diphosphate ligands, see: Ikotun *et al.* (2007 ▶); Marino *et al.* (2008 ▶). Geometric calculatios and checking were performed with *PLATON* (Spek 2009 ▶).

## Experimental

### 

#### Crystal data

Na_2_CaNi_2_(P_2_O_7_)_2_(H_2_O)_10_
*M* *_r_* = 731.48
Orthorhombic, 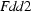

*a* = 11.9340 (11) Å
*b* = 32.774 (4) Å
*c* = 10.9860 (11) Å
*V* = 4296.9 (8) Å^3^
*Z* = 8
Mo *K*α radiation
μ = 2.44 mm^−1^
*T* = 290 K
0.15 × 0.13 × 0.11 mm

#### Data collection

Bruker APEXII CCD diffractometer
Absorption correction: multi-scan (*SADABS*; Bruker, 2001 ▶) *T*_min_ = 0.705, *T*_max_ = 0.772
5540 measured reflections
2085 independent reflections
1999 reflections with *I* > 2σ(*I*)
*R*_int_ = 0.030

#### Refinement

*R*[*F* ^2^ > 2σ(*F* ^2^)] = 0.021
*wR*(*F* ^2^) = 0.049
*S* = 1.07
2085 reflections
150 parameters
1 restraint
H-atom parameters constrained
Δρ_max_ = 0.22 e Å^−3^
Δρ_min_ = −0.29 e Å^−3^
Absolute structure: Flack (1983 ▶), 974 Friedel pairs
Flack parameter: −0.011 (12)

### 

Data collection: *APEX2* (Bruker, 2003 ▶); cell refinement: *SAINT* (Bruker, 2001 ▶); data reduction: *SAINT*; program(s) used to solve structure: *SHELXS97* (Sheldrick, 2008 ▶); program(s) used to refine structure: *SHELXL97* (Sheldrick, 2008 ▶); molecular graphics: *DIAMOND* (Brandenburg & Berndt, 1999 ▶); software used to prepare material for publication: *SHELXL97* and *PLATON* (Spek, 2009 ▶).

## Supplementary Material

Crystal structure: contains datablocks I, global. DOI: 10.1107/S160053681100016X/si2322sup1.cif

Structure factors: contains datablocks I. DOI: 10.1107/S160053681100016X/si2322Isup2.hkl

Additional supplementary materials:  crystallographic information; 3D view; checkCIF report

## Figures and Tables

**Table 1:** Selected bond lengths (Å)

Ca1—O8	2.254 (2)
Ca1—O8^i^	2.254 (2)
Ca1—O6^ii^	2.309 (2)
Ca1—O6^iii^	2.309 (2)
Ca1—O10	2.428 (2)
Ca1—O10^i^	2.428 (2)
Ni1—O4	1.933 (2)
Ni1—O3	1.951 (2)
Ni1—O1	1.957 (2)
Ni1—O2	1.970 (2)
Ni1—O5	2.353 (3)
Na1—O3	2.342 (3)
Na1—O2	2.353 (2)
Na1—O9^iv^	2.423 (3)
Na1—O10	2.450 (3)
Na1—O9	2.489 (4)
Na1—O11	2.902 (3)

Symmetry codes: (i) 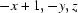; (ii) 
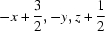
; (iii) 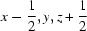; (iv) 
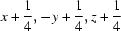
.

**Table 2:** Hydrogen-bond geometry (Å, °)

*D*—H⋯*A*	*D*—H	H⋯*A*	*D*⋯*A*	*D*—H⋯*A*
O5—H52⋯O11	0.94	1.89	2.803 (4)	163
O5—H51⋯O12^v^	0.98	1.96	2.846 (4)	150
O9—H92⋯O4^iii^	0.86	2.14	2.819 (4)	136
O9—H91⋯O11^vi^	0.88	2.35	2.904 (4)	122
O10—H102⋯O1^iii^	0.95	1.75	2.687 (3)	170
O10—H101⋯O12^vii^	0.90	1.89	2.776 (3)	168
O11—H112⋯O8^v^	0.99	1.85	2.834 (3)	175
O11—H111⋯O7^ii^	0.92	2.04	2.953 (3)	173
O12—H122⋯O5^viii^	0.90	2.35	3.121 (4)	143
O12—H121⋯O6	0.93	1.87	2.772 (3)	162

Symmetry codes: (ii) 
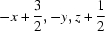
; (iii) 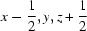; (v) 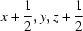; (vi) 
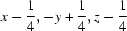
; (vii) 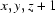; (viii) 
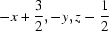
.

**Fig. 1.**
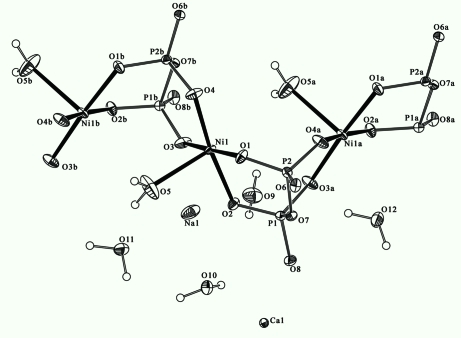
The local coordination feature of the title compound. Displacement ellipsoids are drawn at the 30% probability level (symmetry code: a = -1/4 + x, 1/4 + y, -1/4 + z, b = 1/4 + x, 1/4 - y, 1/4 + z).

**Fig. 2.**
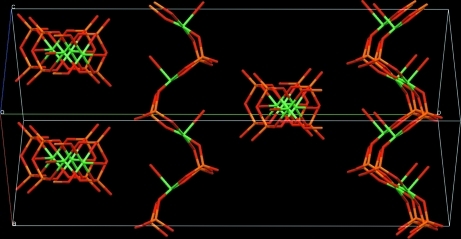
A view of chain construct of the title compound. H, Ca, Na, and O12 atoms are omitted for clarity.

**Fig. 3.**
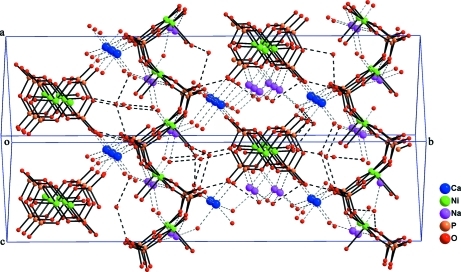
A view of a section of the three-dimensional structure of the title compound. H-bonds are drawn as black dashed lines.

## supplementary crystallographic information

### Comment

The design and synthesis of pyrophosphate-bridged coordination compounds have drawn considerable attention due to its ability to mediate electronic interactions between paramagnetic metal centers. The structure and magnetic properties of a pyrophosphate-bridged Cu(II) complex has been reported by Kruger *et al.* (2001). Two other rare examples of Ni^II^ (Mn^II^) and Co^II^ coordination complexes with pyrophosphate ligands and their magnetic properties have been described by Ikotun *et al.* (2007) and Marino *et al.* (2008).

Wooldridgeite is a hydrated sodium-calcium-copper pyrophosphate mineral, Na_2_CaCu^2+^_2_(P_2_O_7_)_2_(H_2_O)_10_, which is isotypic with the Ni^2+^ title pyrophosphate, described from the Judkins quarry, Nuneaton, Warwickshire, England (Cooper & Hawthorne, 1999). The geometric parameters of the copper mineral and the nickel title hydrate are very similar due to same space group and similar unit cell parameters. The vertex distances in the Wooldridgeite mineral (Cu—O4 and Cu—O4a) are described as 2.37 (2) Å and 3.39 (2) Å, respectively. Those for the nickel isotype are observed as Ni1—O5 = 2.353 (3) Å and Ni1—O5a = 3.355 (3) Å [calculated with PLATON (Spek, 2009)]. Relevant M—O distances (M = Ni, Ca, Na) of the title structure are presented in Table 1.

As shown in Fig. 1, there is one crystallographic Ni^II^ site surrounded by five O atoms in a square pyramidal or strongly distorted (4 + 1 + 1) octahedral arrangement, with Ni—O distances in the range of 1.933 (2) to 2.353 (3) Å, the long Ni—O distance is 3.355 (3) Å. Each Ni octahedron links through one *trans* pair of symmetry related vertices to other Ni octahedra to form a chain, this chain is decorated by [P_2_O_7_] groups to form a chain of octahedra and tetrahedra [Ni(P_2_O_7_)H_2_O]_n_ (Fig. 2). These chains extend along [1 0 1] and [1 0 - 1] and linked into sheets by Na and Ca octahedra, the pattern of chains extending along [1 0 1] (Fig. 3). Together with the coordinated Ca and Na cations, a three-dimensional framework is generated by ten intermolecular O—H···O hydrogen bonds with water and phosphate oxygen atoms as acceptors (Table 2).

### Experimental

A mixture of Na_4_P_2_O_7_ (0.4476 g, 1.0 mmol), Ni(CH_3_COO)_2_ (0.2441 g, 0.98 mmol), Ca(CH_3_COO)_2._H_2_O (0.1763 g, 1.0 mmol), 2,2-Bipyridyl (0.1502 g, 0.96 mmol) and water (15 ml) was adjusted to pH 3.49 with H_2_SO_4_ (1 mol/*L*) solution. The filtration was allowed to stand over several days to give green block single crystals in 42% yield. Elementary Analysis calculated for Na_2_CaNi_2_(P_2_O_7_)_2_(H_2_O)_10_: H 2.76, Ca 5.47, Na 6.29, Ni 16.05, O 52.50, P 16.94%; found: H 2.74, Ca 5.48, Na 6.28, Ni 16.07, O 52.52, P 16.95%.

### Refinement

H atoms of water molecules were located from difference Fourier maps and treated as riding mode with O—H distances in the range of 0.86 to 0.98 Å. All H atoms were allocated displacement parameters related to those of their parent atoms [*U*_iso_(H) = 1.2 *U*_eq_(O)]

### Crystal data

**Table:**

Na_2_CaNi_2_(P_2_O_7_)_2_(H_2_O)_10_	*F*(000) = 2960
*M**_r_* = 731.48	*D*_x_ = 2.261 Mg m^−^^3^
Orthorhombic, *F**d**d*2	Mo *K*α radiation, λ = 0.71073 Å
Hall symbol: F 2 -2d	Cell parameters from 937 reflections
*a* = 11.9340 (11) Å	θ = 1.8–26.5°
*b* = 32.774 (4) Å	µ = 2.44 mm^−^^1^
*c* = 10.9860 (11) Å	*T* = 290 K
*V* = 4296.9 (8) Å^3^	Block, green
*Z* = 8	0.15 × 0.13 × 0.11 mm

### Data collection

**Table:**

Bruker CCD APEXII diffractometer	2085 independent reflections
Radiation source: fine-focus sealed tube	1999 reflections with *I* > 2σ(*I*)
graphite	*R*_int_ = 0.030
ω and φ scans	θ_max_ = 26.0°, θ_min_ = 2.5°
Absorption correction: multi-scan (*SADABS*; Bruker, 2001)	*h* = −9→14
*T*_min_ = 0.705, *T*_max_ = 0.772	*k* = −40→40
5540 measured reflections	*l* = −13→13

### Refinement

**Table:**

Refinement on *F*^2^	Secondary atom site location: difference Fourier map
Least-squares matrix: full	Hydrogen site location: inferred from neighbouring sites
*R*[*F*^2^ > 2σ(*F*^2^)] = 0.021	H-atom parameters constrained
*wR*(*F*^2^) = 0.049	*w* = 1/[σ^2^(*F*_o_^2^) + (0.0191*P*)^2^] where *P* = (*F*_o_^2^ + 2*F*_c_^2^)/3
*S* = 1.07	(Δ/σ)_max_ < 0.001
2085 reflections	Δρ_max_ = 0.22 e Å^−^^3^
150 parameters	Δρ_min_ = −0.29 e Å^−^^3^
1 restraint	Absolute structure: Flack (1983), 974 Friedel pairs
Primary atom site location: structure-invariant direct methods	Flack parameter: −0.011 (12)

### Special details

**Table:**

Geometry. All e.s.d.'s (except the e.s.d. in the dihedral angle between two l.s. planes) are estimated using the full covariance matrix. The cell e.s.d.'s are taken into account individually in the estimation of e.s.d.'s in distances, angles and torsion angles; correlations between e.s.d.'s in cell parameters are only used when they are defined by crystal symmetry. An approximate (isotropic) treatment of cell e.s.d.'s is used for estimating e.s.d.'s involving l.s. planes.
Refinement. Refinement of *F*^2^ against ALL reflections. The weighted *R*-factor *wR* and goodness of fit *S* are based on *F*^2^, conventional *R*-factors *R* are based on *F*, with *F* set to zero for negative *F*^2^. The threshold expression of *F*^2^ > σ(*F*^2^) is used only for calculating *R*-factors(gt) *etc*. and is not relevant to the choice of reflections for refinement. *R*-factors based on *F*^2^ are statistically about twice as large as those based on *F*, and *R*- factors based on ALL data will be even larger.

### Fractional atomic coordinates and isotropic or equivalent isotropic displacement parameters (Å^2^)

**Table:**

	*x*	*y*	*z*	*U*_iso_*/*U*_eq_	
Ca1	0.5000	0.0000	0.62448 (8)	0.01385 (17)	
Ni1	0.86875 (3)	0.110744 (11)	0.47330 (3)	0.01615 (10)	
Na1	0.71250 (14)	0.09782 (5)	0.71990 (15)	0.0492 (4)	
P1	0.64133 (7)	0.07042 (2)	0.42464 (7)	0.01640 (16)	
P2	0.79550 (6)	0.06891 (2)	0.22939 (7)	0.01653 (16)	
O1	0.88590 (18)	0.08175 (6)	0.3187 (2)	0.0249 (5)	
O2	0.73480 (18)	0.07854 (7)	0.51498 (19)	0.0238 (5)	
O3	0.8414 (2)	0.14096 (6)	0.6234 (2)	0.0296 (5)	
O5	0.9793 (3)	0.06393 (10)	0.5826 (3)	0.0684 (11)	
H51	1.0545	0.0553	0.5590	0.082*	
H52	0.9577	0.0581	0.6632	0.082*	
O4	0.9895 (2)	0.14533 (6)	0.4175 (2)	0.0305 (5)	
O6	0.83644 (18)	0.03723 (6)	0.14145 (19)	0.0221 (5)	
O7	0.69943 (17)	0.04685 (6)	0.31024 (19)	0.0180 (4)	
O8	0.55500 (18)	0.04126 (6)	0.4715 (2)	0.0246 (5)	
O9	0.5427 (3)	0.13839 (8)	0.6683 (3)	0.0494 (8)	
H91	0.5308	0.1648	0.6733	0.059*	
H92	0.4990	0.1317	0.7274	0.059*	
O10	0.58195 (19)	0.04368 (6)	0.7792 (2)	0.0275 (5)	
H101	0.6151	0.0351	0.8478	0.033*	
H102	0.5175	0.0595	0.7980	0.033*	
O11	0.8778 (2)	0.03869 (7)	0.8000 (3)	0.0375 (6)	
H111	0.8544	0.0120	0.7960	0.045*	
H112	0.9425	0.0387	0.8564	0.045*	
O12	0.6562 (2)	0.01268 (7)	0.0001 (2)	0.0369 (6)	
H121	0.7193	0.0151	0.0491	0.044*	
H122	0.6269	−0.0124	−0.0117	0.044*	

### Atomic displacement parameters (Å^2^)

**Table:**

	*U*^11^	*U*^22^	*U*^33^	*U*^12^	*U*^13^	*U*^23^
Ca1	0.0133 (4)	0.0136 (4)	0.0147 (4)	0.0007 (3)	0.000	0.000
Ni1	0.01661 (18)	0.01719 (17)	0.01464 (17)	−0.00632 (16)	0.00368 (16)	−0.00529 (14)
Na1	0.0593 (10)	0.0601 (9)	0.0283 (7)	−0.0349 (9)	0.0115 (8)	−0.0071 (8)
P1	0.0166 (4)	0.0160 (3)	0.0166 (4)	−0.0011 (3)	0.0018 (3)	0.0013 (3)
P2	0.0168 (4)	0.0169 (3)	0.0159 (3)	0.0026 (3)	0.0006 (3)	−0.0005 (3)
O1	0.0189 (12)	0.0328 (12)	0.0230 (12)	−0.0051 (10)	0.0009 (10)	−0.0112 (10)
O2	0.0220 (12)	0.0326 (11)	0.0169 (11)	−0.0081 (10)	0.0014 (9)	−0.0028 (9)
O3	0.0385 (14)	0.0263 (11)	0.0241 (12)	−0.0140 (11)	0.0132 (11)	−0.0087 (10)
O5	0.0506 (19)	0.108 (3)	0.0462 (18)	0.042 (2)	0.0165 (15)	0.0406 (18)
O4	0.0346 (14)	0.0267 (11)	0.0301 (13)	−0.0153 (11)	0.0136 (12)	−0.0113 (10)
O6	0.0208 (11)	0.0243 (10)	0.0212 (11)	0.0050 (10)	−0.0013 (9)	−0.0080 (9)
O7	0.0182 (11)	0.0160 (9)	0.0198 (10)	−0.0015 (9)	0.0020 (9)	−0.0029 (8)
O8	0.0219 (11)	0.0281 (11)	0.0238 (11)	−0.0075 (10)	0.0015 (10)	0.0040 (10)
O9	0.066 (2)	0.0474 (16)	0.0347 (14)	−0.0080 (15)	0.0152 (14)	0.0011 (12)
O10	0.0240 (12)	0.0317 (12)	0.0269 (13)	0.0031 (11)	−0.0025 (10)	−0.0016 (10)
O11	0.0377 (15)	0.0262 (11)	0.0487 (17)	−0.0059 (11)	−0.0046 (12)	0.0052 (12)
O12	0.0335 (15)	0.0386 (13)	0.0385 (16)	−0.0031 (12)	−0.0104 (11)	−0.0029 (12)

### Geometric parameters (Å, °)

**Table:**

Ca1—O8	2.254 (2)	Na1—O9^iv^	2.423 (3)
Ca1—O8^i^	2.254 (2)	Na1—O10	2.450 (3)
Ca1—O6^ii^	2.309 (2)	Na1—O9	2.489 (4)
Ca1—O6^iii^	2.309 (2)	Na1—O11	2.902 (3)
Ca1—O10	2.428 (2)	P1—O8	1.497 (2)
Ca1—O10^i^	2.428 (2)	P1—O3^v^	1.508 (2)
Ca1—P2^iii^	3.5191 (8)	P1—O2	1.517 (2)
Ca1—P2^ii^	3.5191 (8)	P1—O7	1.630 (2)
Ca1—Na1	4.2200 (15)	P2—O6	1.500 (2)
Ca1—Na1^i^	4.2200 (15)	P2—O4^v^	1.511 (2)
Ni1—O4	1.933 (2)	P2—O1	1.518 (2)
Ni1—O3	1.951 (2)	P2—O7	1.621 (2)
Ni1—O1	1.957 (2)	P2—Ca1^vi^	3.5191 (8)
Ni1—O2	1.970 (2)	O3—P1^iv^	1.508 (2)
Ni1—O5	2.353 (3)	O4—P2^iv^	1.511 (2)
Ni1—Na1	3.3160 (16)	O6—Ca1^vi^	2.309 (2)
Na1—O3	2.342 (3)	O9—Na1^v^	2.423 (3)
Na1—O2	2.353 (2)		
			
O8—Ca1—O8^i^	83.63 (12)	O5—Ni1—Na1	79.34 (8)
O8—Ca1—O6^ii^	97.52 (8)	O3—Na1—O2	69.82 (9)
O8^i^—Ca1—O6^ii^	89.39 (8)	O3—Na1—O9^iv^	92.02 (10)
O8—Ca1—O6^iii^	89.39 (8)	O2—Na1—O9^iv^	150.00 (11)
O8^i^—Ca1—O6^iii^	97.52 (8)	O3—Na1—O10	167.19 (11)
O6^ii^—Ca1—O6^iii^	170.74 (11)	O2—Na1—O10	97.58 (9)
O8—Ca1—O10	92.92 (7)	O9^iv^—Na1—O10	98.45 (10)
O8^i^—Ca1—O10	173.07 (8)	O3—Na1—O9	96.27 (10)
O6^ii^—Ca1—O10	85.10 (8)	O2—Na1—O9	91.02 (10)
O6^iii^—Ca1—O10	88.42 (7)	O9^iv^—Na1—O9	115.25 (10)
O8—Ca1—O10^i^	173.07 (8)	O10—Na1—O9	85.97 (9)
O8^i^—Ca1—O10^i^	92.92 (7)	O3—Na1—O11	95.40 (10)
O6^ii^—Ca1—O10^i^	88.42 (7)	O2—Na1—O11	91.94 (9)
O6^iii^—Ca1—O10^i^	85.10 (8)	O9^iv^—Na1—O11	65.37 (9)
O10—Ca1—O10^i^	91.12 (11)	O10—Na1—O11	82.41 (8)
O8—Ca1—P2^iii^	93.50 (5)	O9—Na1—O11	168.28 (10)
O8^i^—Ca1—P2^iii^	115.28 (6)	O3—Na1—Ni1	35.32 (6)
O6^ii^—Ca1—P2^iii^	153.99 (6)	O2—Na1—Ni1	35.82 (6)
O6^iii^—Ca1—P2^iii^	17.90 (5)	O9^iv^—Na1—Ni1	119.28 (9)
O10—Ca1—P2^iii^	70.83 (5)	O10—Na1—Ni1	131.87 (8)
O10^i^—Ca1—P2^iii^	82.51 (6)	O9—Na1—Ni1	101.75 (8)
O8—Ca1—P2^ii^	115.28 (6)	O11—Na1—Ni1	87.18 (7)
O8^i^—Ca1—P2^ii^	93.50 (5)	O3—Na1—Ca1	137.79 (8)
O6^ii^—Ca1—P2^ii^	17.90 (5)	O2—Na1—Ca1	68.06 (6)
O6^iii^—Ca1—P2^ii^	153.99 (6)	O9^iv^—Na1—Ca1	127.04 (8)
O10—Ca1—P2^ii^	82.51 (6)	O10—Na1—Ca1	29.95 (6)
O10^i^—Ca1—P2^ii^	70.83 (5)	O9—Na1—Ca1	81.95 (7)
P2^iii^—Ca1—P2^ii^	141.77 (4)	O11—Na1—Ca1	88.65 (6)
O8—Ca1—Na1	63.55 (6)	Ni1—Na1—Ca1	103.42 (4)
O8^i^—Ca1—Na1	144.66 (7)	O8—P1—O3^v^	113.09 (14)
O6^ii^—Ca1—Na1	82.73 (6)	O8—P1—O2	113.15 (13)
O6^iii^—Ca1—Na1	94.96 (6)	O3^v^—P1—O2	112.81 (14)
O10—Ca1—Na1	30.25 (6)	O8—P1—O7	104.81 (11)
O10^i^—Ca1—Na1	121.07 (6)	O3^v^—P1—O7	106.16 (12)
P2^iii^—Ca1—Na1	81.25 (3)	O2—P1—O7	105.95 (12)
P2^ii^—Ca1—Na1	89.39 (3)	O6—P2—O4^v^	113.05 (13)
O8—Ca1—Na1^i^	144.66 (7)	O6—P2—O1	112.14 (12)
O8^i^—Ca1—Na1^i^	63.55 (6)	O4^v^—P2—O1	112.95 (13)
O6^ii^—Ca1—Na1^i^	94.96 (6)	O6—P2—O7	105.94 (11)
O6^iii^—Ca1—Na1^i^	82.73 (6)	O4^v^—P2—O7	106.24 (12)
O10—Ca1—Na1^i^	121.07 (6)	O1—P2—O7	105.81 (12)
O10^i^—Ca1—Na1^i^	30.25 (6)	O6—P2—Ca1^vi^	28.24 (8)
P2^iii^—Ca1—Na1^i^	89.39 (3)	O4^v^—P2—Ca1^vi^	131.10 (9)
P2^ii^—Ca1—Na1^i^	81.25 (3)	O1—P2—Ca1^vi^	84.08 (9)
Na1—Ca1—Na1^i^	151.23 (5)	O7—P2—Ca1^vi^	112.57 (7)
O4—Ni1—O3	95.47 (9)	P2—O1—Ni1	128.42 (13)
O4—Ni1—O1	86.08 (9)	P1—O2—Ni1	122.61 (13)
O3—Ni1—O1	175.96 (11)	P1—O2—Na1	126.15 (13)
O4—Ni1—O2	173.20 (10)	Ni1—O2—Na1	99.80 (9)
O3—Ni1—O2	86.53 (9)	P1^iv^—O3—Ni1	132.45 (14)
O1—Ni1—O2	91.52 (9)	P1^iv^—O3—Na1	126.69 (13)
O4—Ni1—O5	97.25 (11)	Ni1—O3—Na1	100.73 (10)
O3—Ni1—O5	89.63 (11)	P2^iv^—O4—Ni1	129.96 (14)
O1—Ni1—O5	93.88 (11)	P2—O6—Ca1^vi^	133.86 (13)
O2—Ni1—O5	89.25 (10)	P2—O7—P1	120.81 (12)
O4—Ni1—Na1	138.85 (7)	P1—O8—Ca1	147.13 (15)
O3—Ni1—Na1	43.95 (7)	Na1^v^—O9—Na1	128.74 (13)
O1—Ni1—Na1	134.90 (7)	Ca1—O10—Na1	119.80 (10)
O2—Ni1—Na1	44.37 (6)		
			
O4—Ni1—Na1—O3	11.86 (18)	O2—Ni1—O1—P2	40.92 (18)
O1—Ni1—Na1—O3	−174.48 (15)	O5—Ni1—O1—P2	130.27 (18)
O2—Ni1—Na1—O3	−159.31 (16)	Na1—Ni1—O1—P2	51.5 (2)
O5—Ni1—Na1—O3	100.70 (14)	O8—P1—O2—Ni1	173.16 (13)
O4—Ni1—Na1—O2	171.18 (15)	O3^v^—P1—O2—Ni1	−56.86 (19)
O3—Ni1—Na1—O2	159.31 (16)	O7—P1—O2—Ni1	58.88 (17)
O1—Ni1—Na1—O2	−15.16 (14)	O8—P1—O2—Na1	−50.97 (19)
O5—Ni1—Na1—O2	−99.98 (13)	O3^v^—P1—O2—Na1	79.01 (17)
O4—Ni1—Na1—O9^iv^	−31.96 (17)	O7—P1—O2—Na1	−165.25 (12)
O3—Ni1—Na1—O9^iv^	−43.83 (13)	O4—Ni1—O2—P1	23.7 (9)
O1—Ni1—Na1—O9^iv^	141.70 (12)	O3—Ni1—O2—P1	130.99 (16)
O2—Ni1—Na1—O9^iv^	156.86 (14)	O1—Ni1—O2—P1	−45.47 (16)
O5—Ni1—Na1—O9^iv^	56.88 (12)	O5—Ni1—O2—P1	−139.33 (17)
O4—Ni1—Na1—O10	−168.76 (14)	Na1—Ni1—O2—P1	145.2 (2)
O3—Ni1—Na1—O10	179.38 (18)	O4—Ni1—O2—Na1	−121.5 (7)
O1—Ni1—Na1—O10	4.90 (17)	O3—Ni1—O2—Na1	−14.22 (11)
O2—Ni1—Na1—O10	20.07 (13)	O1—Ni1—O2—Na1	169.32 (10)
O5—Ni1—Na1—O10	−79.92 (15)	O5—Ni1—O2—Na1	75.45 (11)
O4—Ni1—Na1—O9	96.08 (14)	O3—Na1—O2—P1	−130.91 (17)
O3—Ni1—Na1—O9	84.22 (13)	O9^iv^—Na1—O2—P1	173.25 (18)
O1—Ni1—Na1—O9	−90.26 (12)	O10—Na1—O2—P1	51.46 (17)
O2—Ni1—Na1—O9	−75.10 (12)	O9—Na1—O2—P1	−34.60 (16)
O5—Ni1—Na1—O9	−175.08 (11)	O11—Na1—O2—P1	134.06 (15)
O4—Ni1—Na1—O11	−91.57 (14)	Ni1—Na1—O2—P1	−143.5 (2)
O3—Ni1—Na1—O11	−103.44 (13)	Ca1—Na1—O2—P1	46.30 (13)
O1—Ni1—Na1—O11	82.09 (12)	O3—Na1—O2—Ni1	12.57 (9)
O2—Ni1—Na1—O11	97.25 (11)	O9^iv^—Na1—O2—Ni1	−43.3 (2)
O5—Ni1—Na1—O11	−2.73 (11)	O10—Na1—O2—Ni1	−165.06 (10)
O4—Ni1—Na1—Ca1	−179.51 (11)	O9—Na1—O2—Ni1	108.87 (10)
O3—Ni1—Na1—Ca1	168.63 (13)	O11—Na1—O2—Ni1	−82.47 (10)
O1—Ni1—Na1—Ca1	−5.85 (11)	Ca1—Na1—O2—Ni1	−170.23 (10)
O2—Ni1—Na1—Ca1	9.31 (9)	O4—Ni1—O3—P1^iv^	3.7 (2)
O5—Ni1—Na1—Ca1	−90.67 (10)	O1—Ni1—O3—P1^iv^	−108.7 (13)
O8—Ca1—Na1—O3	−21.31 (14)	O2—Ni1—O3—P1^iv^	−169.8 (2)
O8^i^—Ca1—Na1—O3	2.33 (19)	O5—Ni1—O3—P1^iv^	100.9 (2)
O6^ii^—Ca1—Na1—O3	80.87 (14)	Na1—Ni1—O3—P1^iv^	175.9 (3)
O6^iii^—Ca1—Na1—O3	−108.14 (14)	O4—Ni1—O3—Na1	−172.19 (12)
O10—Ca1—Na1—O3	173.6 (2)	O1—Ni1—O3—Na1	75.5 (14)
O10^i^—Ca1—Na1—O3	164.60 (14)	O2—Ni1—O3—Na1	14.33 (11)
P2^iii^—Ca1—Na1—O3	−119.68 (13)	O5—Ni1—O3—Na1	−74.94 (12)
P2^ii^—Ca1—Na1—O3	97.53 (13)	O2—Na1—O3—P1^iv^	171.1 (2)
Na1^i^—Ca1—Na1—O3	167.93 (14)	O9^iv^—Na1—O3—P1^iv^	−33.4 (2)
O8—Ca1—Na1—O2	−25.22 (9)	O10—Na1—O3—P1^iv^	−178.3 (5)
O8^i^—Ca1—Na1—O2	−1.58 (13)	O9—Na1—O3—P1^iv^	82.3 (2)
O6^ii^—Ca1—Na1—O2	76.96 (9)	O11—Na1—O3—P1^iv^	−98.82 (18)
O6^iii^—Ca1—Na1—O2	−112.05 (9)	Ni1—Na1—O3—P1^iv^	−176.2 (3)
O10—Ca1—Na1—O2	169.71 (14)	Ca1—Na1—O3—P1^iv^	167.22 (12)
O10^i^—Ca1—Na1—O2	160.69 (9)	O2—Na1—O3—Ni1	−12.72 (10)
P2^iii^—Ca1—Na1—O2	−123.59 (7)	O9^iv^—Na1—O3—Ni1	142.82 (11)
P2^ii^—Ca1—Na1—O2	93.62 (7)	O10—Na1—O3—Ni1	−2.1 (6)
Na1^i^—Ca1—Na1—O2	164.03 (7)	O9—Na1—O3—Ni1	−101.50 (11)
O8—Ca1—Na1—O9^iv^	−175.17 (13)	O11—Na1—O3—Ni1	77.37 (12)
O8^i^—Ca1—Na1—O9^iv^	−151.54 (13)	Ca1—Na1—O3—Ni1	−16.59 (19)
O6^ii^—Ca1—Na1—O9^iv^	−72.99 (12)	O3—Ni1—O4—P2^iv^	4.3 (2)
O6^iii^—Ca1—Na1—O9^iv^	97.99 (12)	O1—Ni1—O4—P2^iv^	−179.5 (2)
O10—Ca1—Na1—O9^iv^	19.75 (14)	O2—Ni1—O4—P2^iv^	111.1 (8)
O10^i^—Ca1—Na1—O9^iv^	10.73 (13)	O5—Ni1—O4—P2^iv^	−86.0 (2)
P2^iii^—Ca1—Na1—O9^iv^	86.45 (11)	Na1—Ni1—O4—P2^iv^	−4.0 (3)
P2^ii^—Ca1—Na1—O9^iv^	−56.33 (11)	O4^v^—P2—O6—Ca1^vi^	135.88 (17)
Na1^i^—Ca1—Na1—O9^iv^	14.07 (10)	O1—P2—O6—Ca1^vi^	6.8 (2)
O8—Ca1—Na1—O10	165.08 (13)	O7—P2—O6—Ca1^vi^	−108.17 (17)
O8^i^—Ca1—Na1—O10	−171.28 (15)	O6—P2—O7—P1	174.81 (14)
O6^ii^—Ca1—Na1—O10	−92.74 (13)	O4^v^—P2—O7—P1	−64.71 (18)
O6^iii^—Ca1—Na1—O10	78.24 (13)	O1—P2—O7—P1	55.59 (17)
O10^i^—Ca1—Na1—O10	−9.02 (17)	Ca1^vi^—P2—O7—P1	145.68 (11)
P2^iii^—Ca1—Na1—O10	66.71 (11)	O8—P1—O7—P2	177.56 (14)
P2^ii^—Ca1—Na1—O10	−76.08 (11)	O3^v^—P1—O7—P2	57.63 (18)
Na1^i^—Ca1—Na1—O10	−5.68 (11)	O2—P1—O7—P2	−62.54 (17)
O8—Ca1—Na1—O9	69.16 (9)	O3^v^—P1—O8—Ca1	−132.5 (2)
O8^i^—Ca1—Na1—O9	92.80 (12)	O2—P1—O8—Ca1	−2.6 (3)
O6^ii^—Ca1—Na1—O9	171.34 (9)	O7—P1—O8—Ca1	112.4 (2)
O6^iii^—Ca1—Na1—O9	−17.68 (9)	O8^i^—Ca1—O8—P1	−141.1 (3)
O10—Ca1—Na1—O9	−95.92 (13)	O6^ii^—Ca1—O8—P1	−52.6 (3)
O10^i^—Ca1—Na1—O9	−104.93 (9)	O6^iii^—Ca1—O8—P1	121.2 (2)
P2^iii^—Ca1—Na1—O9	−29.21 (7)	O10—Ca1—O8—P1	32.8 (2)
P2^ii^—Ca1—Na1—O9	−172.00 (7)	O10^i^—Ca1—O8—P1	158.4 (6)
Na1^i^—Ca1—Na1—O9	−101.60 (7)	P2^iii^—Ca1—O8—P1	103.8 (2)
O8—Ca1—Na1—O11	−117.86 (9)	P2^ii^—Ca1—O8—P1	−50.3 (3)
O8^i^—Ca1—Na1—O11	−94.22 (12)	Na1—Ca1—O8—P1	25.4 (2)
O6^ii^—Ca1—Na1—O11	−15.68 (8)	Na1^i^—Ca1—O8—P1	−162.32 (17)
O6^iii^—Ca1—Na1—O11	155.31 (9)	O3—Na1—O9—Na1^v^	64.92 (15)
O10—Ca1—Na1—O11	77.06 (12)	O2—Na1—O9—Na1^v^	−4.89 (15)
O10^i^—Ca1—Na1—O11	68.05 (10)	O9^iv^—Na1—O9—Na1^v^	160.14 (15)
P2^iii^—Ca1—Na1—O11	143.77 (7)	O10—Na1—O9—Na1^v^	−102.41 (16)
P2^ii^—Ca1—Na1—O11	0.98 (7)	O11—Na1—O9—Na1^v^	−109.5 (5)
Na1^i^—Ca1—Na1—O11	71.38 (7)	Ni1—Na1—O9—Na1^v^	29.56 (15)
O8—Ca1—Na1—Ni1	−31.08 (7)	Ca1—Na1—O9—Na1^v^	−72.56 (14)
O8^i^—Ca1—Na1—Ni1	−7.44 (12)	O8—Ca1—O10—Na1	−13.35 (11)
O6^ii^—Ca1—Na1—Ni1	71.10 (7)	O8^i^—Ca1—O10—Na1	46.6 (7)
O6^iii^—Ca1—Na1—Ni1	−117.91 (7)	O6^ii^—Ca1—O10—Na1	83.96 (12)
O10—Ca1—Na1—Ni1	163.84 (13)	O6^iii^—Ca1—O10—Na1	−102.65 (12)
O10^i^—Ca1—Na1—Ni1	154.83 (7)	O10^i^—Ca1—O10—Na1	172.28 (15)
P2^iii^—Ca1—Na1—Ni1	−129.45 (4)	P2^iii^—Ca1—O10—Na1	−106.04 (11)
P2^ii^—Ca1—Na1—Ni1	87.76 (4)	P2^ii^—Ca1—O10—Na1	101.78 (11)
Na1^i^—Ca1—Na1—Ni1	158.16 (5)	Na1^i^—Ca1—O10—Na1	176.81 (6)
O6—P2—O1—Ni1	−163.26 (14)	O3—Na1—O10—Ca1	−19.7 (6)
O4^v^—P2—O1—Ni1	67.6 (2)	O2—Na1—O10—Ca1	−9.63 (13)
O7—P2—O1—Ni1	−48.21 (19)	O9^iv^—Na1—O10—Ca1	−164.18 (11)
Ca1^vi^—P2—O1—Ni1	−160.03 (16)	O9—Na1—O10—Ca1	80.87 (12)
O4—Ni1—O1—P2	−132.71 (19)	O11—Na1—O10—Ca1	−100.59 (11)
O3—Ni1—O1—P2	−20.1 (15)	Ni1—Na1—O10—Ca1	−21.31 (17)

Symmetry codes: (i) −*x*+1, −*y*, *z*; (ii) −*x*+3/2, −*y*, *z*+1/2; (iii) *x*−1/2, *y*, *z*+1/2; (iv) *x*+1/4, −*y*+1/4, *z*+1/4; (v) *x*−1/4, −*y*+1/4, *z*−1/4; (vi) *x*+1/2, *y*, *z*−1/2.

### Hydrogen-bond geometry (Å, °)

**Table:**

*D*—H···*A*	*D*—H	H···*A*	*D*···*A*	*D*—H···*A*
O5—H52···O11	0.94	1.89	2.803 (4)	163
O5—H51···O12^vii^	0.98	1.96	2.846 (4)	150
O9—H92···O4^iii^	0.86	2.14	2.819 (4)	136
O9—H91···O11^v^	0.88	2.35	2.904 (4)	122
O10—H102···O1^iii^	0.95	1.75	2.687 (3)	170
O10—H101···O12^viii^	0.90	1.89	2.776 (3)	168
O11—H112···O8^vii^	0.99	1.85	2.834 (3)	175
O11—H111···O7^ii^	0.92	2.04	2.953 (3)	173
O12—H122···O5^ix^	0.90	2.35	3.121 (4)	143
O12—H121···O6	0.93	1.87	2.772 (3)	162

Symmetry codes: (vii) *x*+1/2, *y*, *z*+1/2; (iii) *x*−1/2, *y*, *z*+1/2; (v) *x*−1/4, −*y*+1/4, *z*−1/4; (viii) *x*, *y*, *z*+1; (ii) −*x*+3/2, −*y*, *z*+1/2; (ix) −*x*+3/2, −*y*, *z*−1/2.
